# Bringing Back a Scientific and Updated Approach to Wildlife Conservation: A Response. Reply to Beltrán, J.F.; Rodríguez-Rodríguez, E.J. Relying on Incomplete Information Can Lead to the Wrong Conclusions. Comment on “van Hassel, F.; Bovenkerk, B. How Should We Help Wild Animals Cope with Climate Change? The Case of the Iberian Lynx. *Animals* 2023, *13*, 453”

**DOI:** 10.3390/ani14020184

**Published:** 2024-01-05

**Authors:** Falco van Hassel, Bernice Bovenkerk

**Affiliations:** Philosophy Group, Department of Communication, Philosophy, Technology, and Education (CPTE), Wageningen University and Research, Hollandseweg 1, 6706 KN Wageningen, The Netherlands

We are pleased that our paper on the need to extend climate justice to animals [1] was read and critically examined by researchers from Universidad de Sevilla; we thank the authors for their comments.

In their comment “Bringing Back a Scientific and Updated Approach to Wildlife Conservation” on “How Should We Help Wild Animals Cope with Climate Change? The Case of the Iberian Lynx” Beltrán and Rodriguez-Rodriguez [2] correctly point out that the Iberian Lynx (*Lynx pardinus*) population has risen over the last decade due to intensive conservation efforts. We agree with their statement that the Iberian lynx is a good example of successful conservation biology. Our paper should in no way be seen as a critique on the conservation measures that have already been taken. In our paper, we tried to clearly introduce the case of the Iberian lynx by referring to recent developments and research. Nevertheless, we also considered that this species was used as an illustrative case, and that our paper is not intended as a complete assessment of the robustness of the Iberian lynx’s population. Therefore, we chose not to discuss the current status of the Iberian lynx in too much detail. As a result, as mentioned by Beltrán and Rodriguez-Rodriguez, some recent papers discussing the Iberian lynx were not included.

The purpose of our paper is to argue that it is essential to consider how we should help wild animals cope with climate change. We have taken the Iberian lynx as an illustrative case to be able to extrapolate this argument to a broader ethical discussion. While the severity of future climate events is uncertain, it is clear that wild animals are already affected by climate change and will continue to be in the future. Therefore, we think that a discussion on conservation biology in relation to climate change is necessary.

We are still convinced that the Iberian lynx is a suitable case study for our paper. While the population of the species has risen, this unfortunately does not mean that the population is no longer prone to extinction due to climate change, especially when looking at the long-term future. Even when the population had risen in the past, different researchers discussed climate change as a major threat to the survival of the species [3,4]. The fact that the population has continued to increase has likely made the species more robust towards the effects of climate change. However, this does not imply that the species’ long-term survival is now secured. Climate change and related changes in weather conditions will affect the habitat of the Iberian lynx, with, among others, an expected increase in temperature, droughts, precipitation extremes and heatwaves, resulting in changes in the ecosystem, and influencing the Iberian lynx [5,6,7,8]. This threat is also confirmed by the IUCN [9].

Additionally, Beltrán and Rodriguez-Rodriguez mention that the conservation measures we propose are not original or new. We do not claim these measures to be original or new, and it was also not our intention to propose new conservation methods. In contrary, as written in our paper, we wanted to examine the moral implications of intervention strategies. Logically, we chose strategies that have already been applied or are being discussed.

We hope that with this reply, we have clarified the purpose of our paper and the importance of climate justice for animals, also in relation to the Iberian lynx.
